# Supplementary material for: A Comprehensive Assessment of Ultraviolet-Radiation-Induced Mutations in Flammulina filiformis Using Whole-Genome Resequencing
Source: J Fungi (Basel). 2024 Mar 20;10(3):228. doi: 10.3390/jof10030228 (PMC10971301; doi:10.3390/jof10030228)
Supplement: Supplementary file 1 [file jof-10-00228-s001.zip › Supplementary Material S8/KEGG annotation/out/64381550635650.os/KO/out_map/map04916.html]

KEGG PATHWAY: Melanogenesis - Reference pathway


|  |  |
| --- | --- |
| **Melanogenesis - Reference pathway** |  |

[
Pathway menu
| Organism menu
| Pathway entry
| Show description
| User data mapping
]

Cutaneous melanin pigment plays a critical role in camouflage, mimicry, social communication, and protection against harmful effects of solar radiation. Melanogenesis is under complex regulatory control by multiple agents. The most important positive regulator of melanogenesis is the MC1 receptor with its ligands melanocortic peptides. MC1R activates the cyclic AMP (cAMP) response-element binding protein (CREB). Increased expression of MITF and its activation by phosphorylation (P) stimulate the transcription of tyrosinase (TYR), tyrosinase-related protein 1 (TYRP1), and dopachrome tautomerase (DCT), which produce melanin. Melanin synthesis takes place within specialized intracellular organelles named melanosomes. Melanin-containing melanosomes then move from the perinuclear region to the dendrite tips and are transferred to keratinocytes by a still not well-characterized mechanism.

|  |  |
| --- | --- |
| Reference pathway | 100% |
